# Molecular mechanism of central nervous system repair by the *Drosophila* NG2 homologue *kon-tiki*

**DOI:** 10.1083/jcb.201603054

**Published:** 2016-08-29

**Authors:** Maria Losada-Perez, Neale Harrison, Alicia Hidalgo

**Affiliations:** NeuroDevelopment Lab, School of Biosciences, University of Birmingham, Birmingham B15 2TT, England, UK

## Abstract

Neuron glia antigen 2 (NG2)–positive glia are repair cells that proliferate upon central nervous system (CNS) damage, promoting functional recovery. However, repair is limited because of the failure of the newly produced glial cells to differentiate. It is a key goal to discover how to regulate NG2 to enable glial proliferation and differentiation conducive to repair. *Drosophila* has an *NG2* homologue called *kon-tiki* (*kon*), of unknown CNS function. We show that *kon* promotes repair and identify the underlying mechanism. Crush injury up-regulates *kon* expression downstream of *Notch.* Kon in turn induces glial proliferation and initiates glial differentiation by activating glial genes and *prospero* (*pros*). Two negative feedback loops with Notch and Pros allow Kon to drive the homeostatic regulation required for repair. By modulating Kon levels in glia, we could prevent or promote CNS repair. Thus, the functional links between Kon, Notch, and Pros are essential for, and can drive, repair. Analogous mechanisms could promote CNS repair in mammals.

## Abstract (precis)

Glial cells help central nervous system injury repair, but this is limited by the failure of newly produced glial cells to differentiate. Here, Losada-Perez et al. identify the NG2-dependent mechanism modulating glial proliferation and differentiation after damage to promote repair, in the central nervous system of *Drosophila*.

## Introduction

Injury to the central nervous system (CNS) induces a glial regenerative response (GRR) that is evolutionarily conserved across animals, from flies to humans (Smith et al., 1987; Franklin and ffrench-Constant, 2008; Kato et al., 2011). Ensheathing glia proliferate and remyelinate axons, and phagocytic cells clear up the cell debris caused by the injury, together leading to partial functional recovery (Franklin and ffrench-Constant, 2008). Regeneration is limited partly because the newly generated ensheathing glial cells can fail to differentiate. Also, axon growth inhibitors accumulate, and astrocyte activation creates a scar, which contains the lesion but inhibits axonal regrowth (Yiu and He, 2006; Barres, 2008; Sofroniew, 2009). Also, the inflammatory response activates macrophages and microglia, which clear the cell debris but also attack myelin and provoke cell death (Aguzzi et al., 2013). In the mammalian CNS, the proliferative GRR is performed by oligodendrocyte progenitor cells (OPCs), which express neuron glia antigen 2 (NG2; Franklin and ffrench-Constant, 2008; Trotter et al., 2010; Zuo and Nishiyama, 2013). NG2^+^ OPCs (or NG2 cells) maintain CNS integrity and homeostasis: They interact closely with synapses; express factors involved in neurotransmitter recycling and neurotransmitter receptors; are the main CNS cell population to continuously divide throughout life; produce trophic factors that sustain neuronal survival; and are the progenitor cells for oligodendrocytes (OLs) that myelinate CNS axons (Franklin and ffrench-Constant, 2008; Trotter et al., 2010; Zuo and Nishiyama, 2013). NG2^+^ OPCs are the first cell type to divide upon injury and drive spontaneous remyelination and are permissive to neurite growth (Franklin and ffrench-Constant, 2008). Transplantation of CNS-ensheathing glia to the site of spinal cord injury can promote partial recovery of locomotion in humans (Tabakow et al., 2014). Conversely, dysregulation of NG2^+^ OPC proliferation causes brain tumors, and NG2 is a glioma marker (Persson et al., 2010). Unfortunately, GRR does not necessarily result in differentiation of the newly generated glial cells. A key challenge is to find out how to control NG2^+^ OPC proliferation and OL differentiation to drive regeneration and restore structural homeostasis.

NG2 is a transmembrane protein with a large extracellular domain, which contains two N-terminal laminin neurexin sex-hormone globulin motifs, and an intracellular PDZ domain (Trotter et al., 2010). Cleavage by α- and γ-secretases results in four protein products, including a secreted form, and an intracellular domain, which regulates gene expression (Trotter et al., 2010). NG2 is not expressed in neurons or astrocytes, is expressed in all OPCs but not differentiated OLs, and is expressed in pericytes and macrophages/microglia (Cahoy et al., 2008; Kucharova and Stallcup, 2010; Trotter et al., 2010). NG2 is required for OPC proliferation in development and structural homeostasis (Kucharova and Stallcup, 2010). CNS injury induces the up-regulation of NG2 and NG2^+^ OPC proliferation (Kucharova et al., 2011). NG2-knockout mice have reduced OPC proliferation in development and after injury (Kucharova et al., 2011). Furthermore, whereas in normal animals the size of demyelinating lesions decreases over time as the CNS tends to repair naturally, demyelinating lesions in *NG2* knockout mice fail to shrink, as a result of reduced OPC proliferation and the consequent depletion in OLs (Kucharova et al., 2011). Thus, NG2 is a critical factor underlying the regenerative response of OPCs.

Notch1 maintains OPCs in a proliferative state and inhibits OL differentiation (Wang et al., 1998; Genoud et al., 2002). Notch1 levels in OPCs also increase upon injury, correlating with OPC proliferation and remyelination in mice. However, remyelination fails as many newly generated OPCs do not differentiate into ensheathing OLs, presumably because of high Notch levels (Franklin and ffrench-Constant, 2008). To understand and promote repair, it is essential to find out what genes might work with and counteract *Notch*.

*Drosophila* is a powerful model organism to discover gene networks and investigate CNS injury, regeneration, and repair (MacDonald et al., 2006; Ayaz et al., 2008; Xiong et al., 2010; Kato et al., 2011; Song et al., 2012). *Drosophila melanogaster* has an *NG2* homologue, called *kon-tiki* (*kon*) or *perdido* (Estrada et al., 2007; Schnorrer et al., 2007). The extracellular domains of NG2 and Kon are highly conserved, and so is the intracellular PDZ motif (Stegmüller et al., 2003; Estrada et al., 2007; Schnorrer et al., 2007). Kon has not been investigated in the CNS.

The *Drosophila* GRR to the injured ventral nerve cord (VNC; equivalent to the spinal cord) is performed by a subset of CNS neuropil glial (NG) cells (Kato et al., 2011) that express *prospero* (*pros*; hereafter referred to as Pros^+^ NG). Pros^+^ NG have the nuclei and main cell bodies outside the axonal neuropil and extend fine cytoplasmic projections both around and into the neuropil, interacting closely with synapses (Kato et al., 2011; Stork et al., 2014; Peco et al., 2016). Pros^+^ NG, also known as astrocytes (Stork et al., 2014; Peco et al., 2016), also enwrap the entire neuropil bundle, individual axons, and clusters of smaller axons (Kato et al., 2011). In the normal larva, Pros^+^ NG do not divide and are quiescent in G1 (Kato et al., 2011). Injury to the larval CNS provokes NG activation, as they enlarge their cytoplasms, become phagocytic, clear the cell debris, engulf and dissolve vacuoles, and proliferate and differentiate, promoting reenwrapment and CNS repair (Kato et al., 2011).

Pros is a homeodomain transcription factor that inhibits glial cell division and promotes glial differentiation (Griffiths and Hidalgo, 2004; Kato et al., 2011). Pros is required for axonal enwrapment and activates the expression of Ebony and glutamine synthetase 2 (GS2), which are involved in the recycling of dopamine and glutamate, respectively (Griffiths and Hidalgo, 2004; Kato et al., 2011; Peco et al., 2016). Pros also maintains the expression of Notch, an activator of glial cell division, keeping NG in cell-cycle quiescence, ready to respond to damage (Kato et al., 2011). Manipulating the levels of Pros and Notch in NG can switch the response to injury from prevention to promotion of repair (Kato et al., 2011). Whether Kon is involved, and how, is unknown. Here, we ask whether the *Drosophila NG2* homologue *kon* functions in CNS glia and in response to injury.

## Results

### *kon-tiki* is expressed in CNS glial cells

**Figure 1.**
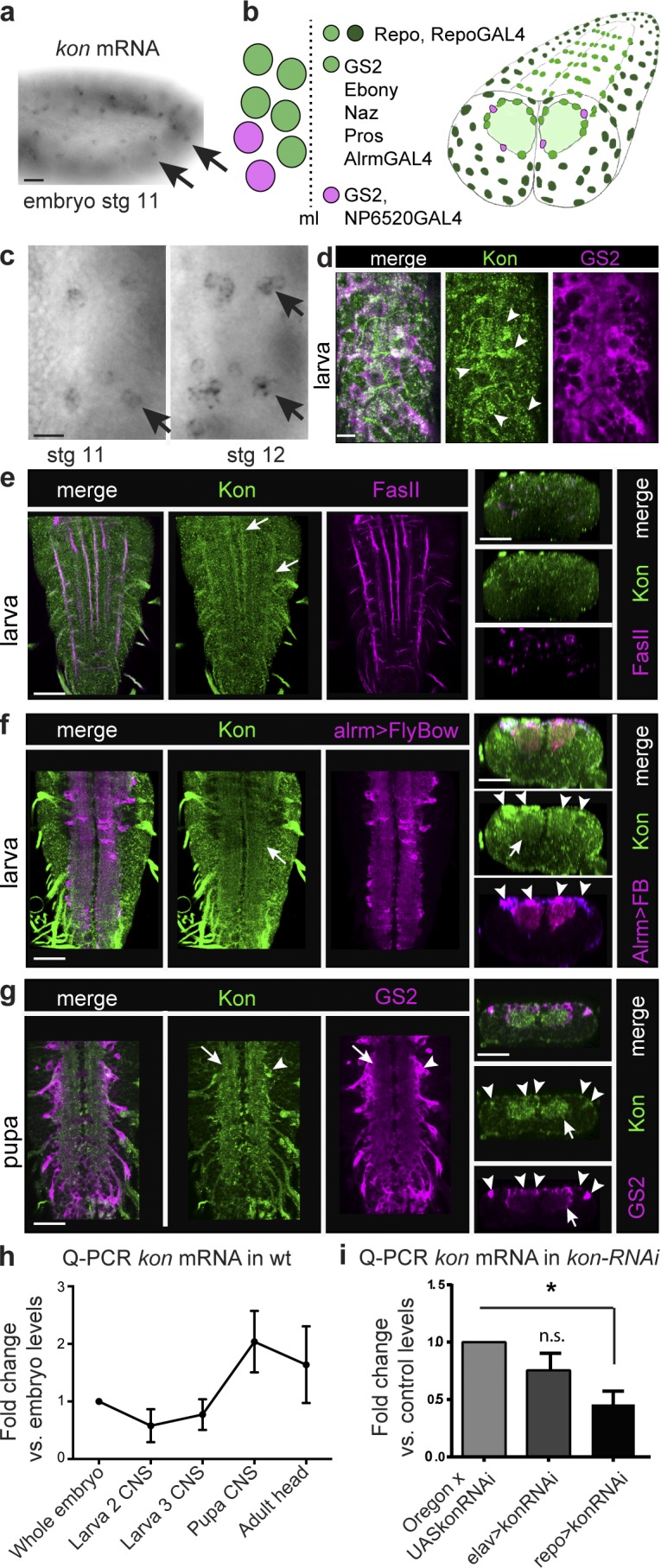
***kon* expression in the CNS.** (a and c) *kon* mRNA distributed in lateral cells in the embryo, including the longitudinal glioblast (arrows), which divides into three to four progeny cells at stage 12 as it migrates into the CNS. (b) Larval abdominal VNC glia. Repo is in all glia except midline glia; the NG are GS2^+^ and comprise the alrmGAL4^+^ Pros^+^ and the NP6520GAL4 Pros^−^ glia; NG in one hemisegment are represented on the left. ElavGAL4 drives expression in all neurons. ml, midline. (d) Anti-Kon colocalized with GS2 in glia (arrowheads) enwrapping the neuropil (horizontal view). (e–g) Anti-Kon in VNC, horizontal views on the left, transverse views on the right. (e) Early, Kon is distributed along FasII^+^ axonal fascicles (arrows). (f) Later, Kon colocalizes with *alrmGAL4>UASFlyBow1.1* within (arrows) and around (arrows) the neuropil. (g) In pupae, Kon is prominent within the neuropil (arrows) and also colocalizes with GS2 in cell bodies wrapping the neuropil (arrowheads). (h and i) qRT-PCR. (h) Wild-type (wt) profile of *kon* transcript levels, in whole embryos, dissected CNS from larvae, pupae, and adult heads, all normalized to embryonic levels. *n* = 3 replicates per time point. Error bars indicate SD. (i) *kon* RNAi knockdown in all glia caused a significant reduction in *kon* transcript levels in wandering larva. *n* = 3 replicates per genotype. One-way ANOVA, P < 0.05; post hoc Sidak test, *, P < 0.05. n.s., not significant. For sample sizes and statistics details, see Table S1; >, GAL4/UAS. Bars: (a) 20 µm; (c and d) 10 µm; (e–g) 50 µm.

*kon* transcripts were found in stage 11 embryos in segmentally repeated groups of cells, including the longitudinal glioblast (LGB; Fig. 1, a and c), the precursor of the NG cells of the VNC (Fig. 1 b). At stage 12, *kon* transcripts were present in three to four cells per hemisegment, consistent with being LGB lineage daughter cells (Fig. 1 c). We did not detect *kon* mRNA in the late embryonic or larval VNC in situ.

Using anti-Kon antibodies to the transmembrane motif, Kon was detected at low levels in the VNC of third-instar larvae, initially colocalizing with FasII along axonal tracts (Fig. 1 e) and subsequently throughout the neuropil (Fig. 1 f). Signal increased at pupa, prominently in the neuropil (Fig. 1 g). The neuropil is formed of axons, dendrites, and cytoplasmic projections from the NG, as visualized with *alrmGAL4>UASFlyBow* (Fig. 1 f). AlrmGAL4^+^ cells are the Pros^+^ NG (Fig. 1 b; Kato et al., 2011). Thus, signal within the neuropil could correspond to neuronal or Pros^+^ NG processes. Using the glial marker GS2, which is found in all VNC and PNS glia enwrapping the neuropil, we detected colocalization with Kon in NG of larvae and pupae (Fig. 1, d and g). These data show that *kon* may be expressed in neurons and is expressed in glial cells.

To further verify whether *kon* was expressed in neurons or glia, we first characterized its developmental expression profile using quantitative real-time RT-PCR (qRT-PCR) in whole embryos and dissected CNS from larvae, pupae, and adult fly heads (Fig. 1 h). *kon* was expressed in embryos, and expression decreased in the larval CNS, before sharply increasing in pupae and adult heads (Fig. 1 h). Next, we knocked down *kon* expression in neurons or glia and measured how this affected overall *kon* mRNA levels in the wandering larval CNS. *kon* knockdown in all neurons (*elavGAL4>UASkon-RNAi^106680^*) did not significantly decrease *kon* mRNA levels compared with control (*UASkonRNAi^106680/+^*; Fig. 1 i). However, *kon* knockdown in all glia except the midline glia (*repoGAL4>UASkonRNAi^106680^*) decreased *kon* mRNA to half the normal levels (Fig. 1 i). *ElavGAL4* is the main neuronal driver in *Drosophila*, but it is transiently expressed in all glia (Berger et al., 2007). Together, these data show that *kon* is prominently expressed in CNS glia. Altogether, these data show that *kon* is expressed in glia, including Pros^+^ GS2^+^ neuropil glia of the VNC.

### Kon promotes glial proliferation

**Figure 2.**
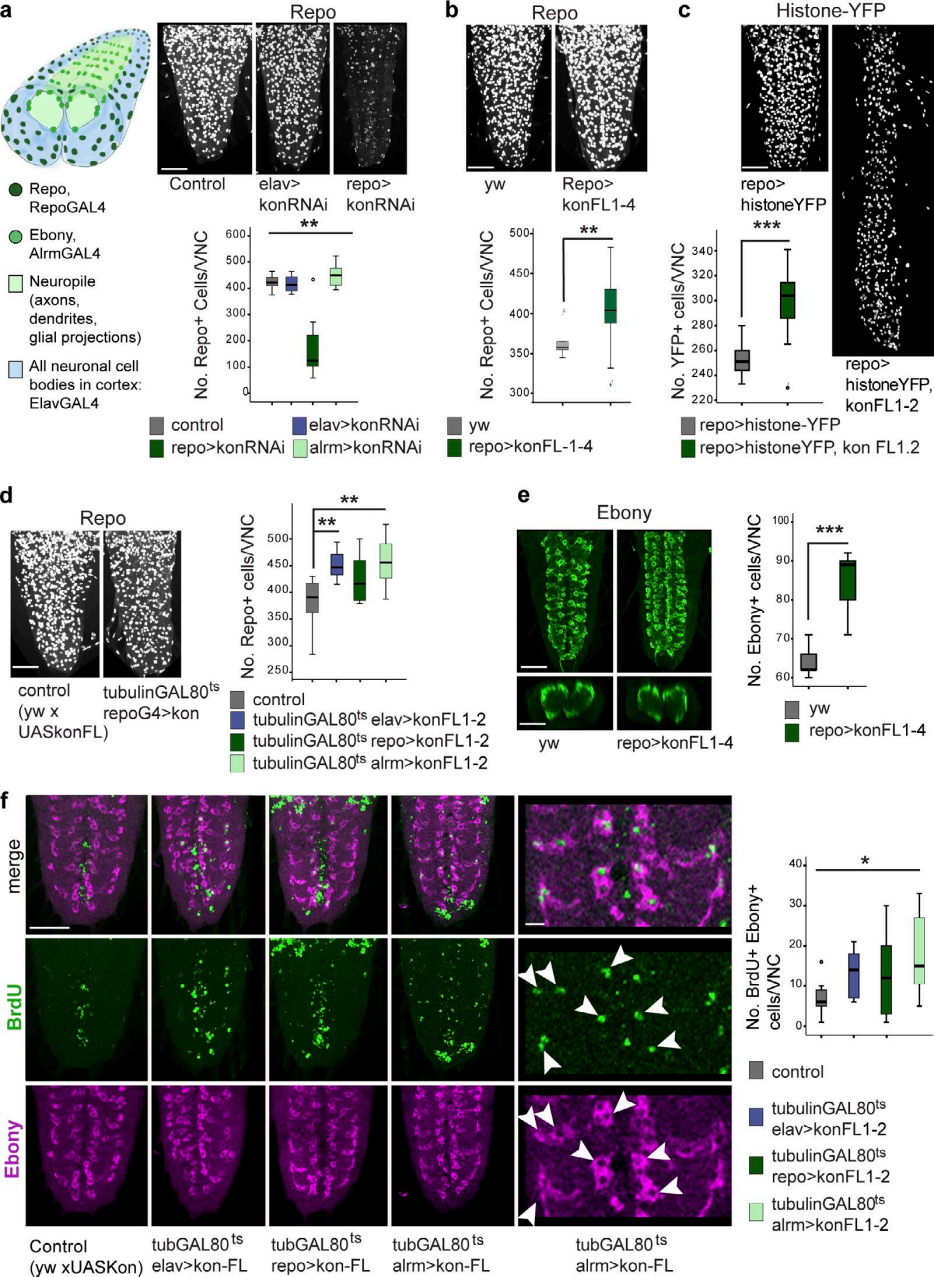
**Kon promotes glial proliferation.** (a–d) Automatic counting of Repo^+^ or YFP^+^ cells in vivo, throughout the abdominal CNS in 3D using DeadEasy Larval Glia software. (a) Drawing represents the larval VNC: neuronal somata (blue) occupy all nonglial space in cortex; glial nuclei within cortex shown in dark green. *kon* knockdown with RNAi in all glia (with *repoGAL4*) reduces Repo^+^ glial number. Kruskal–Wallis: **, P < 0.005. (b) Overexpression of *kon* in all glia increases Repo^+^ glial number. Mann–Whitney *U* test: **, P < 0.01. (c) Overexpression of *kon* in all glia increases YFP reporter glial number. Student’s *t* test: ***, P < 0.000. (d) Temporal restriction of *kon* overexpression to the larva only, in neurons, all glia, and only Pros^+^ alrmGAL4^+^ glia, increased Repo^+^ glial cell number. One-way ANOVA, post hoc Dunnett comparisons to control: **, P < 0.01. (e) Overexpression of *kon* in all glia increased Ebony^+^ NG cell number. Student’s *t* test: ***, P < 0.001. (f) The number of Ebony^+^ NG that incorporated the S-phase marker BrdU (arrowheads) increased when *kon* was overexpressed, particularly in Pros^+^ alrmGAL4^+^ NG. Welch’s ANOVA: *, P < 0.05. Sample types are larval VNCs, *n* = 6–24 per genotype. For details on sample sizes and statistics, see Table S1; > denotes GAL4/UAS. Bars: (a–e) 50 µm; (f) 10 µm.

To ask what functions Kon might have in glia, as homozygous null *kon* mutants are lethal in the embryo, we tested the effects of *kon* knockdown using RNAi. The total number of Repo^+^ glial cells in third-instar larval VNCs were counted in vivo automatically using DeadEasy Larval Glia software (Forero et al., 2012). *kon* knockdown in all neurons (*elavGAL4>UASkonRNAi^106680^*) or only the Pros^+^ NG (*alrmGAL4>konRNAi^106680^*), did not affect total glial cell number (Fig. 2 a). However, *kon* knockdown in all glia (*repoGAL4>UASkonRNAi^106680^*) decreased Repo^+^ glial cell number compared with controls (Fig. 2 a). Thus, *kon* is required in glia for normal cell number. In the larva, *alrmGAL4* is expressed in only a small fraction of the total Repo^+^ glia, and because all Repo^+^ cells were counted, the effect of *alrmGAL4* knockdown may be undetectable with this test. Furthermore, NG divide in embryos but not appreciably in larvae (Griffiths and Hidalgo, 2004; Kato et al., 2011), and *alrmGAL4* is not expressed during the embryonic divisions of the NG cell lineage. *RepoGAL4* is expressed throughout development, including in the embryonic LGB. Thus, the observed decrease in larval glial cell number with *repoGAL4* but not *alrmGAL4* could be caused by interference with the divisions of the LGB. In fact, overexpression of *kon* in all glia throughout development (*repoGAL4>UASkon*), caused a significant increase in Repo^+^ glial cell number (Fig. 2 b). This was confirmed using a second marker, histone-YFP (*repoGAL4>UASHistone-YFP*, *UASKon*; Fig. 2 c), as the number of YFP^+^ glia increased when *kon* was overexpressed. These data suggest that Kon promotes glial proliferation. To test whether Kon could promote glial proliferation specifically in larvae, we restricted *kon* overexpression to larval stages using *tubulin-GAL80^ts^.* GAL80^ts^ represses *GAL4* expression at 18°C but not at 30°C; thus we kept *GAL4* turned off during embryogenesis. Overexpression of *kon*, in larva only, in all neurons (*tubulin-GAL80^ts^*, *elavGAL4>UASkon*), all glia (*tubulin-GAL80^ts^*, *repoGAL4>UASkon*), or NG only (*tubulin-GAL80^ts^*, *alrmGAL4>UASkon*), increased Repo^+^ glial cell number. This suggests that *kon* is sufficient to activate glial proliferation in larvae (Fig. 2 d). Importantly, the number of NG glial cells also increased when *kon* was overexpressed in glia (*repoGAL4>UASkon*), as detected with Ebony, a downstream target of Pros and marker for the AlrmGAL4^+^ NG (Griffiths and Hidalgo, 2004; Kato et al., 2011; Peco et al., 2016; Fig. 2 e). Altogether, these data suggest that Kon is required for and can induce glial proliferation, including of NG cells.

To test whether the increase in cell number observed with *kon* overexpression was caused by increased cell division, we performed a BrdU pulse experiment. BrdU incorporates into DNA in the S phase of the cell cycle, and when applied in a pulse reveals cells undergoing cell-cycle progression at that time point. Overexpression of *kon* was restricted to 24 h in the third-instar larval stage: starting with a 6-h BrdU pulse, cell cycle progression was enabled for a further 18 h, after which the larval CNSs were dissected. Overexpression of *kon* in third-instar larvae, in all neurons (*tubulinGAL80^ts^ elavGAL4>UASkon*), all glia (*tubulinGAL80^ts^ repoGAL4>UASkon*), or NG only (*tubulinGAL80^ts^ alrmGAL4>UASkon*), increased BrdU incorporation in Ebony^+^ NG cells (Fig. 2 f). These data demonstrate that Kon induces NG cell division.

### Kon regulates glial marker expression and cell shape

**Figure 3.**
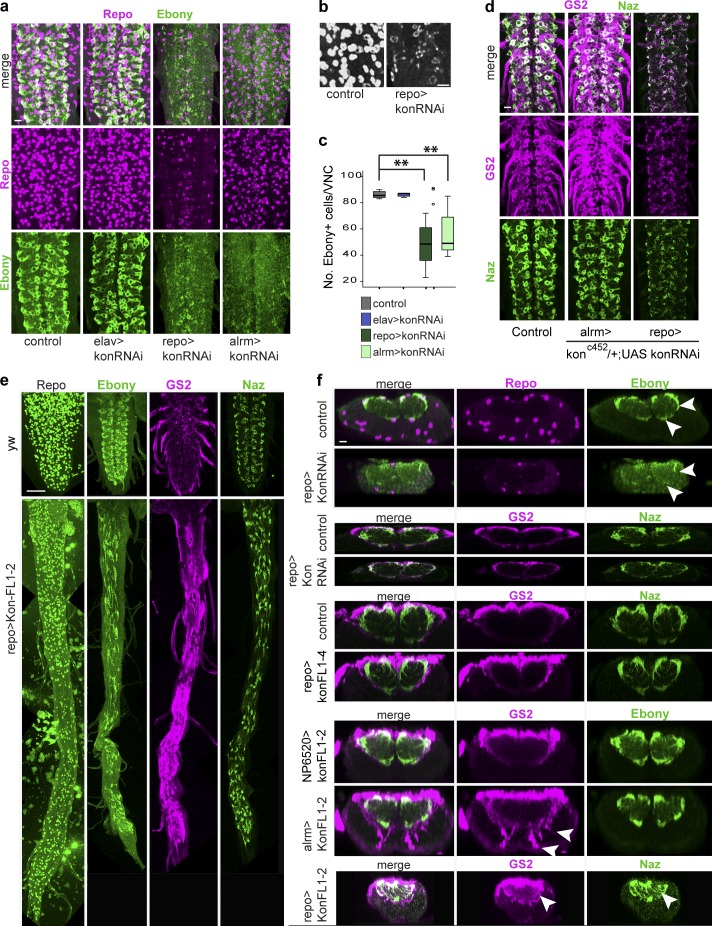
**Kon influences glial marker expression and glial shape.** (a–c and f) *kon* knockdown in glia with RNAi down-regulated Repo and Ebony (arrowheads in f) and decreased the number of Ebony^+^ NG. Notice perinuclear Repo in panel b. (c) Welch’s ANOVA: P = 0.000, post hoc Games–Howell comparisons to control: **, P < 0.05. (d) *kon* knockdown in glia with RNAi down-regulated GS2 and Naz. (e and f) Overexpression of *kon* using line *UASkonFL1-2* elongated the VNC and up-regulated Ebony, GS2, and Naz. (f) *kon* overexpression with *alrmGAL4* and *repoGAL4* up-regulated GS2 and Naz within the neuropil and in the cortex (arrowheads), but with *NP6520* had no effect. Horizontal views in a, b, d, and e; transverse views in f. Sample types are larval VNCs. (c) *n* = 8–9 per genotype. For further details, see Table S1. >, GAL4/UAS. Bars: (a, b, d, and f) 10 µm; (e) 50 µm.

To ask whether Kon might influence glial differentiation, we visualized the effect of altering *kon* levels on the expression of NG markers. *kon* knockdown in all neurons (*elavGAL4>UASkonRNAi^106680^*) had no effect on glial Repo or Ebony levels (Fig. 3 a). However, *kon* knockdown in all glia (*repoGAL4>UASkonRNAi^106680^*) decreased Repo and Ebony and reduced the number of Ebony^+^ NG (Fig. 3, a–c; and Fig. S1 a). Repo was distributed in perinuclear rings, rather than filling the glial nuclei (Fig. 3 b). *kon RNAi* in Pros^+^ NG (with *alrmGAL4*) did not affect Repo but caused loss of Ebony and reduced Ebony^+^ NG cell number (Fig. 3, a and c). The glial markers GS2 and Naz were not affected by *kon* knockdown in Pros^+^ NG, even when using a stronger condition in a *kon^C452^*-null heterozygous background (*kon^C452/+^; alrmGAL4/UASkonRNAi*; Fig. 3 d). However, both Naz and GS2 were dramatically down-regulated with *repoGAL4* knockdown (*kon^C452/+^; repoGAL4/UASkonRNAi*; Fig. 3, d and f). Because *repoGAL4* but not *alrmGAL4* is expressed from the early development of the glia, these data imply that Kon is required for the onset of glial differentiation, but not the maintenance—except for maintaining Ebony.

To test whether overexpression of *kon* affected glial markers, we used two *UASkon* inserts. Overexpression of *kon* in all glia (*repoGAL4*) with *UASkonFL1-2,* but not *UASkonFL1-4,* caused extremely long VNCs (Fig. 3 e). This is caused by interference with embryogenesis, because when *kon* was overexpressed in the larval period only, using GAL80^ts^, VNCs were not as long (Fig. 2 d). More relevantly, overexpression of *kon* in all glia with the weaker line (*repoGAL4>UASkonFL1-4*) increased levels of the NG marker GS2 surrounding the neuropil and thickened the Naz projections into the neuropil (Fig. 3 f). With the stronger line *UASkonFL1-2,* overexpression in Pros-negative NG (*NP6520>UASkonFL1-2*) did not affect Ebony or GS2 (Fig. 3 f). However, overexpression in the Pros^+^ NG (*alrmGAL4>UASkonFL1-2*) increased GS2 and induced glial projections with high GS2 levels both within the neuropil and in the cortex (Fig. 3 f). GS2 signal is normally weak within the neuropil and absent from the cortex. Overexpression with *repoGAL4>UASkonFL1-2* caused a dramatic up-regulation of both GS2 and Naz within the neuropil (Fig. 3, e and f) and an apparent change in cell shape, as glia showed more Naz^+^ filopodia (Fig. S1 b). These data show that Kon positively regulates the glial differentiation markers GS2 and Naz. Altogether, these data show that *kon* is required for the onset of glial differentiation, to positively regulate the glial markers Repo, Ebony, GS2, and Naz, but Kon is not sufficient to drive glial differentiation and maintenance outside Pros^+^ NG.

### Kon is functionally linked to Pros and Notch

NG differentiation and proliferation depend on Pros and Notch (Griffiths and Hidalgo, 2004; Griffiths et al., 2007; Kato et al., 2011; Peco et al., 2016). Thus, we next asked whether *kon* might be functionally related to *pros* or *Notch.*

**Figure 4.**
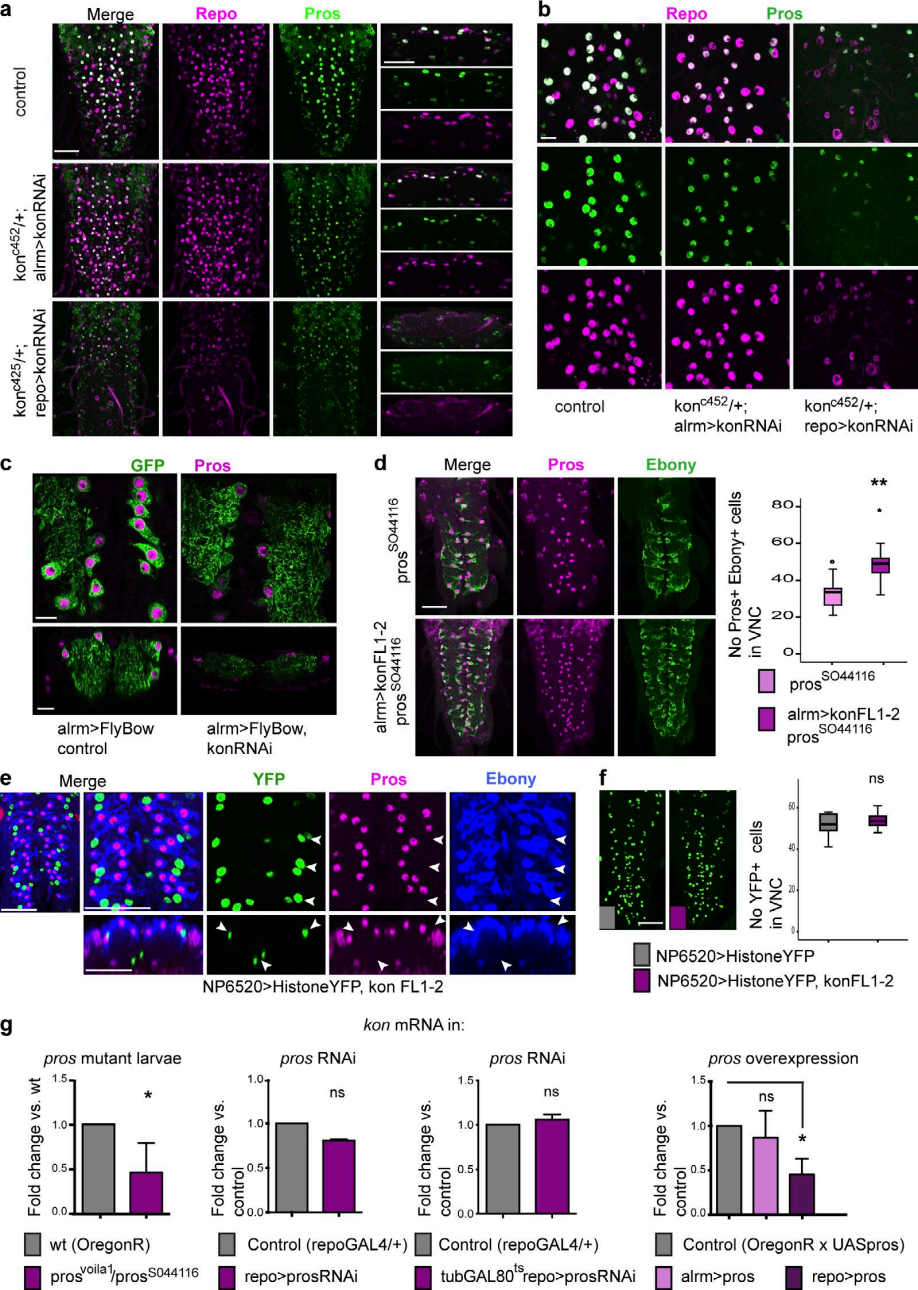
**Kon is functionally related to Pros.** (a and b) *kon* RNAi knockdown down-regulated Repo and Pros in NG. (a) Horizontal views on the left and transverse views on the right. (c) *kon* RNAi knockdown down-regulated Pros; glia visualized with Flybow. (d) Overexpression of *kon* rescued the hypomorphic *pros^S044116^* homozygous mutant phenotype in Pros^+^ Ebony^+^ NG. Student’s *t* test: **, P < 0.005. (e and f) Overexpression of *kon* with *NP6520* in Pros-negative glia did not affect Pros or Ebony (e, arrowheads) and did not induce proliferation (f). Student’s *t* test: ns, not significant. (g) qRT-PCR showing *kon* mRNA levels, which decreased in *pros* loss-of-function mutant larvae (Student’s *t* test: *, P < 0.05) but not with *pros* RNAi knockdown in glia throughout development, or restricted to a larval time window; Student’s *t* test: P = 0.111. *kon* expression decreased with the overexpression of *pros* in all glia. One-way ANOVA: *, P < 0.05. Sample types are larval VNCs. (d) *n* = 9–12; (f) *n* = 10–12; (g) qRT-PCR *n* = 20 per genotype per replicate and three replicates per genotype. For further details, see Table S1. >, GAL4/UAS. Bars: (a and d–f) 50 µm; (b and c) 10 µm.

Pros inhibits NG proliferation and activates the expression of glial differentiation markers *ebony* and *GS2* (Griffiths and Hidalgo, 2004; Kato et al., 2011; Peco et al., 2016). *kon* knockdown with *alrmGAL4>UASkonRNAi^106680^* down-regulated Pros and Ebony and decreased the number of Pros^+^ Ebony^+^ NG (Fig. S2 a), as was also observed with FlyBow (*alrmGAL4>UASFB1.1*, *UASkonRNAi^106680^*; Fig. 4 c). To test for a stronger effect, we targeted *konRNAi* to glia in a heterozygous *kon^c452^*-null mutant background (*kon^c452/+^*; *repoGAL4/UASkon-RNAi*)*.* This caused the dramatic down-regulation of Repo and Pros (Fig. 4, a and b). Importantly, all cells with low Repo also had low Pros. This means that Kon is required for *repo* and *pros* expression and glial differentiation. Overexpression of *kon* in glia (*repoGAL4>UASkon*) had no apparent effect on glial Pros (Fig. S2 b). Thus we used hypomorphic *pros^S044116^* mutants, which have few Pros^+^ Ebony^+^ NG compared with wild-type, to ask whether overexpression of *kon* would alter their incidence. Overexpression of *kon* in *pros^S04416^* glia (*UASKonFL1.2/^+^*; *pros^S044116^ alrmGAL4/pros^S044116^*) rescued the *pros* mutant phenotype, causing an increase in the number of Pros^+^ Ebony^+^ NG (Fig. 4 d). Together, these data show that Pros and its downstream target Ebony decrease with *kon* loss of function and increase with *kon* gain of function, demonstrating that Kon activates Pros expression in NG.

However, *kon* overexpression in *Pros*-negative glia (*NP6520>UASHistoneYFP*, *UASKon*) did not cause the up-regulation of Ebony or Pros (Fig. 4 e). This means that Kon is not sufficient to induce glial differentiation. Similarly, *NP6520>UASHistoneYFP*, *UASKon* did not increase glial cell number either (Fig. 4 f), meaning that Kon is not sufficient to induce glial proliferation. Together, these data imply that Kon function is restricted to Pros^+^ and alrm^+^ NG and is tightly linked to Notch and Pros.

To investigate the effects that altering the function of Pros might have on *kon* expression, we used qRT-PCR. *kon* mRNA levels in the third-instar larval CNS were reduced in *pros^volia1^*/*pros^S044116^* mutants compared with wild type (Fig. 4 g), suggesting that Pros activates *kon* expression. However, Pros is required not only in glia, but also in neuroblasts, ganglion mother cells, and neurons; thus *kon* mRNA levels reflect the pleiotropic loss of *pros* function in all these cell types. *pros* knockdown in glia (*repoGAL4>UASpros-RNAi*) did not cause a significant reduction in *kon* mRNA levels (Fig. 4 g), and *pros* knock-down in glia restricted to the larval time window (*tubulinGAL80^ts^*; *repoGAL4>UASprosRNAi*) did not affect *kon* expression either (Fig. 4 g). In contrast, overexpression of *pros* in all glia (*repoGAL4>UASpros*) decreased *kon* transcript levels. Together, these data show that Pros represses *kon* expression.

To conclude, Kon activates *pros* expression, and *pros* represses *kon*. Pros is required for glial differentiation (Griffiths and Hidalgo, 2004; Kato et al., 2011; Peco et al., 2016); thus by activating *pros* as well as glial differentiation markers, Kon initiates glial differentiation in daughter cells. Kon promotes and Pros inhibits cell proliferation; thus by activating its own inhibitor, Kon exerts negative feedback, restoring cell number homeostasis.

**Figure 5.**
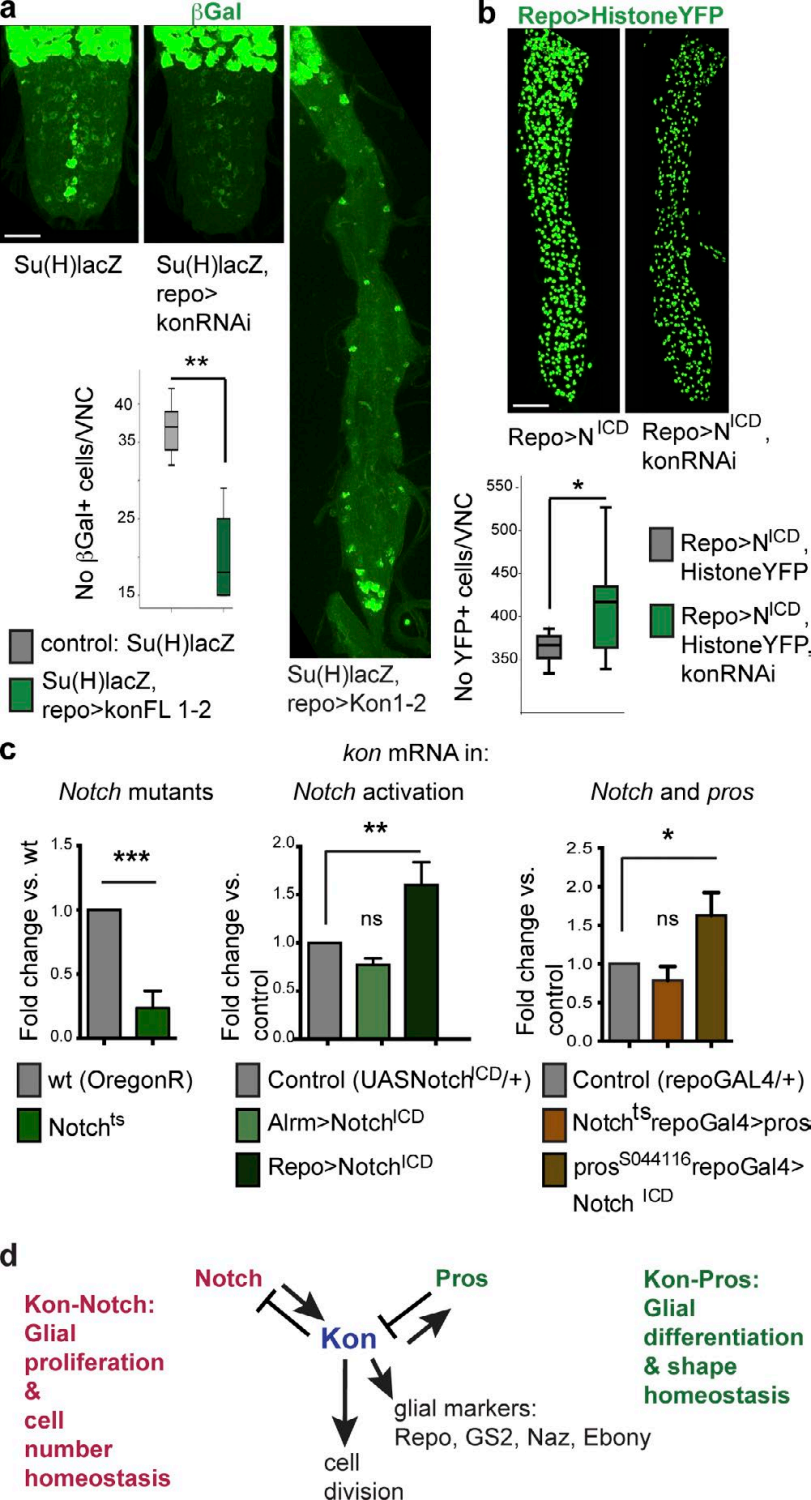
**Kon is functionally related to Notch.** (a) *kon* knockdown in glia did not affect Notch signaling, visualized with the Su(H)lacZ reporter. Overexpression of *kon* in glia caused a reduction in the number of abdominal anti-βGal^+^ cells in larvae. Student’s *t* test: **, P < 0.01. (b) *kon* knockdown in all glia enhanced the increase in glial cell number caused by the overexpression of activated *Notch^ICD^*. Student’s *t* test: *, P < 0.05. (c) qRT-PCR revealing *kon* expression: *kon* expression decreased in *Notch^ts^* mutant male hemizygous larvae (Student’s *t* test: ***, P < 0.001) and increased upon the overexpression of activated *Notch^ICD^* in glia (one-way ANOVA, post hoc Dunnett comparison to control: **, P < 0.01); overexpression of activated *Notch^ICD^* in glia in a *pros^S044116^* mutant background increased *kon* expression; Student’s *t* test: **, P < 0.05. ns, not significant. (d) Diagram illustrating the functional genetic relationships between *kon*, *Notch*, and *pros* in development. (a and b) Sample types are larval VNCs; (a) *n* = 4, 6; (b) *n* = 13, 13. (c) Sample types are dissected CNS; *n* = 20 CNS per genotype per replicate, three replicates per genotype. For further details, see Table S1; >, GAL4/UAS. Bars, 50 µm.

Notch promotes glial proliferation (Griffiths and Hidalgo, 2004; Kato et al., 2011). *kon* knockdown in glia did not affect the expression of the Notch signaling reporter *Su(H)lacZ* (*Su(H)lacZ*, *repoGAL4>UASkonRNAi^106680^*) compared with controls (Fig. 5 a). However, overexpression of *kon* in glia significantly decreased the number of βGAL^+^ cells in the abdominal VNC (Fig. 5 a; *Su(H)lacZ*, *repoGAL4>UASkon*). This means that *kon* represses Notch signaling. βGAL^+^ cell number was reduced despite the overall increase in glial number, implying that Kon can activate glial cell division independently of Notch. To ask whether Notch^ICD^ depends on Kon to activate glial cell proliferation, we tested whether *kon* knockdown in glia could rescue the glial overproliferation phenotype caused by activated Notch^ICD^ (*UAShistoneYFP*; *repoGAL4>UASNotch^ICD^*, *UASkonRNAi* vs. *UAShistoneYFP*; *repoGAL4>UASNotch^ICD^*; Fig. 5 b). *kon* RNAi did not rescue and instead enhanced the phenotype, resulting in an even higher increase in glial cell number (Fig. 5 b). Accordingly, Kon represses Notch signaling. This phenotype is also reminiscent of the synergistic increase in glial cell number when *Notch^ICD^* is overexpressed in *pros* mutants (Kato et al., 2011) and is consistent with *kon* knockdown causing the down-regulation of Pros. Either way, *kon* overexpression decreases Notch signaling, and *kon* knockdown enhances Notch signaling, implying that *kon* represses *Notch.*

To investigate what effects altering the functions of Notch might have on *kon* expression, we used qRT-PCR. In *Notch^ts^* mutants, *kon* mRNA levels were reduced (Fig. 5 c). Conversely, activation of Notch signaling in glia (*repoGAL4>UASNotch^ICD^*) increased *kon* transcript levels (Fig. 5 c). Thus, Notch signaling activates *kon* expression.

Notch and Pros positively regulate each other: in *pros* mutants, Notch signaling is down-regulated, and in *Notch* mutants, Pros is down-regulated (Griffiths and Hidalgo, 2004; Kato et al., 2011). Thus, we asked whether Pros or Notch could regulate *kon* directly. Overexpression of *pros* in glia of *Notch* mutants (*Notch^ts^*; *repo>pros*) resulted in normal *kon* transcript levels, but activating *Notch^ICD^* in glia of *pros* mutants (*Notch^ICD/+^*; *pos^S044116^/pros^S044116^*, *repoGAL4*) increased *kon* mRNA levels (Fig. 5 c). These data suggest that *pros* is not required, but Notch is sufficient, to activate *kon* expression. Therefore, Notch directly activates Kon.

To conclude, Notch signaling activates *kon* expression, and Kon promotes cell proliferation (Fig. 5 d). After cell division, Kon activates glial markers, inducing the onset of glial cell differentiation, and activates *pros,* which maintains glial differentiation. Structural homeostasis comes about with negative feedback, as *kon* represses *Notch* and *pros* represses *kon*, restoring quiescence in daughter cells and cell number homeostasis.

### Crushing injury in living larvae induces a glial regenerative response

**Figure 6.**
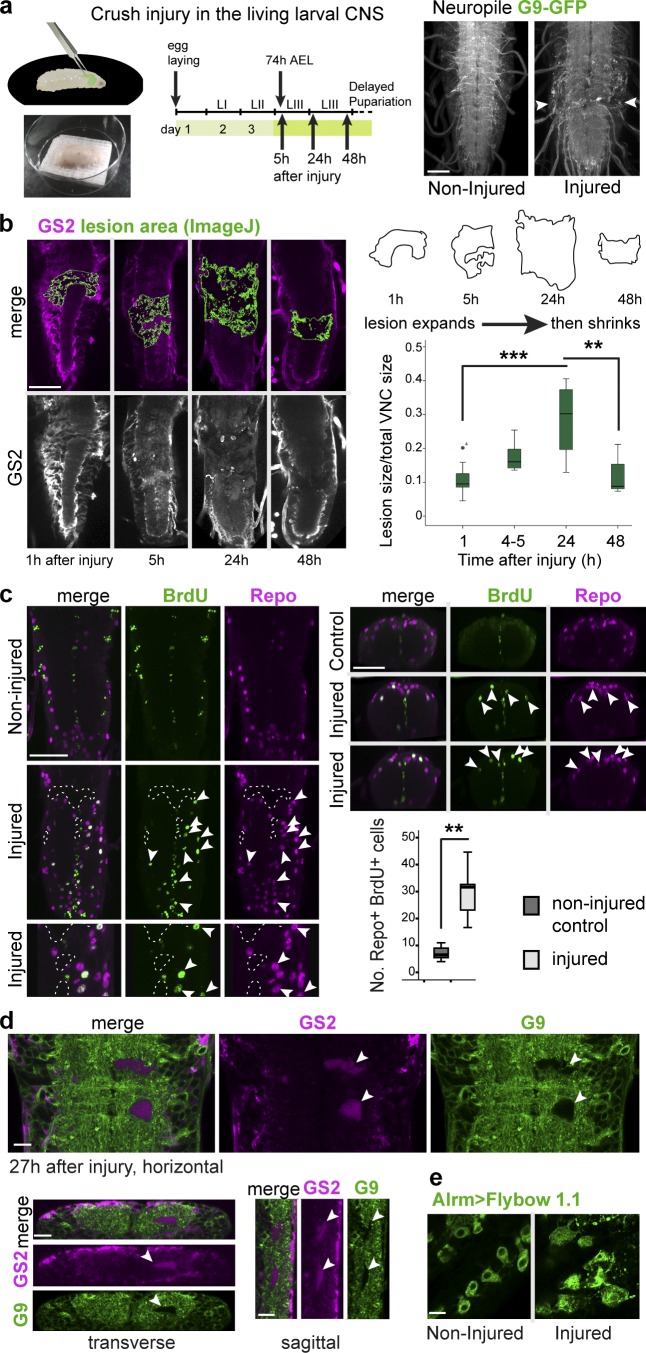
**Crush injury in living larvae induces the glial regenerative response.** (a) Illustration of crushing injury in living, undissected larvae carrying the G9-GFP CNS reporter. Arrowheads in image on the right indicate typical lesion. (b) Crush injury induces a typical progression of wound expansion followed by shrinkage. The lesions are indicated in green. One-way ANOVA, post hoc multiple comparisons Bonferroni test: ***, P < 0.001; **, P < 0.01. (c) Crush injury induced the incorporation of the S-phase marker BrdU into Repo^+^ NG cells (arrowheads). Mann–Whitney *U* test: **, P < 0.005. Horizontal views on the left, transverse views on the right. (d and e) Crush injury induced glial activation. (d) Neuropil holes were invaded by GS2^+^ glial processes (arrowheads). (e) NG enlarged and acquired thicker projections. (b and c) Sample types are larval VNCs; (b) *n* = 4–11; (c) *n* = 8 and 7. For further details, see Table S1; >, GAL4/UAS. Bars: (b and c) 50 µm; (d and e) 10 µm.

To test whether Kon is involved in GRR, we developed a novel method of crushing injury in the living larval CNS (Fig. 6 a). The CNS was visualized with GFP using the G9 exon-trap reporter, which reveals all CNS axons (Fig. 6 a). Crushing injury was applied to the larval VNC under UV light, with a swift closing of fine forceps tips on the VNC (Fig. 6 a), and larvae were kept alive for up to 2 d after injury.

Crush injury induced a GRR. First, we measured lesion progression over time (Fig. 6 b). CNS injury in *Drosophila* and mammals follows a stereotypic progression: the lesion expands first and subsequently shrinks, reflecting a natural tendency to repair (Kato et al., 2011; Kucharova et al., 2011; Kato and Hidalgo, 2013). We measured wound size using anti-GS2, a strong marker of neuropil enwrapment, at 1, 5, 24, and 48 h after injury (Fig. 6 b and Materials and methods). By 24 h after crushing, the mean lesion size had expanded significantly. By 48 h, mean lesion size had shrunk compared with 24-h lesions. These results show that, like stabbing injury (Kato et al., 2011; Kato and Hidalgo, 2013), crush injury induces a natural repair response.

Second, we tested whether crushing injury induced glial proliferation (Fig. 6 c). In intact larva, glial cells do not normally divide and are quiescent in G1, and stabbing injury induces glial proliferation (Kato et al., 2011). To test whether crushing injury induced glial cell division, we performed a 6-h BrdU pulse to visualize cell-cycle progression after injury. This revealed a significant increase in BrdU incorporation in Repo^+^ glial cells in crushed larval VNCs compared with intact controls (Fig. 6 c). Thus, crush injury induces glial cell division.

Third, we tested whether crush injury induced glial activation (Fig. 6 d). Stabbing injury caused NG to become phagocytic, extending large cytoplasmic projections that engulfed cellular debris, enwrapped and dissolved vacuoles caused by the injury, and invaded neuropil holes to invariably repair them (Kato et al., 2011). We visualized glial projections with anti-GS2 and the FlyBow reporter (*alrmGAL4>UASFlyBow1.1*). Crush injury also caused the formation of holes, which were invariably filled by GS2^+^ glial processes (Fig. 6 d). Furthermore, NG cells changed shape upon crush injury, enlarging their cytoplasms and extending multiple projections (Fig. 6 e). Thus, crush injury induces glial activation.

Altogether, these data demonstrate that crushing injury induces a GRR. Wound size initially expands and then shrinks, as the NG change morphology, invade the injury site and neuropil holes, and proliferate, altogether enabling repair.

### Kon promotes CNS repair after crushing injury

**Figure 7.**
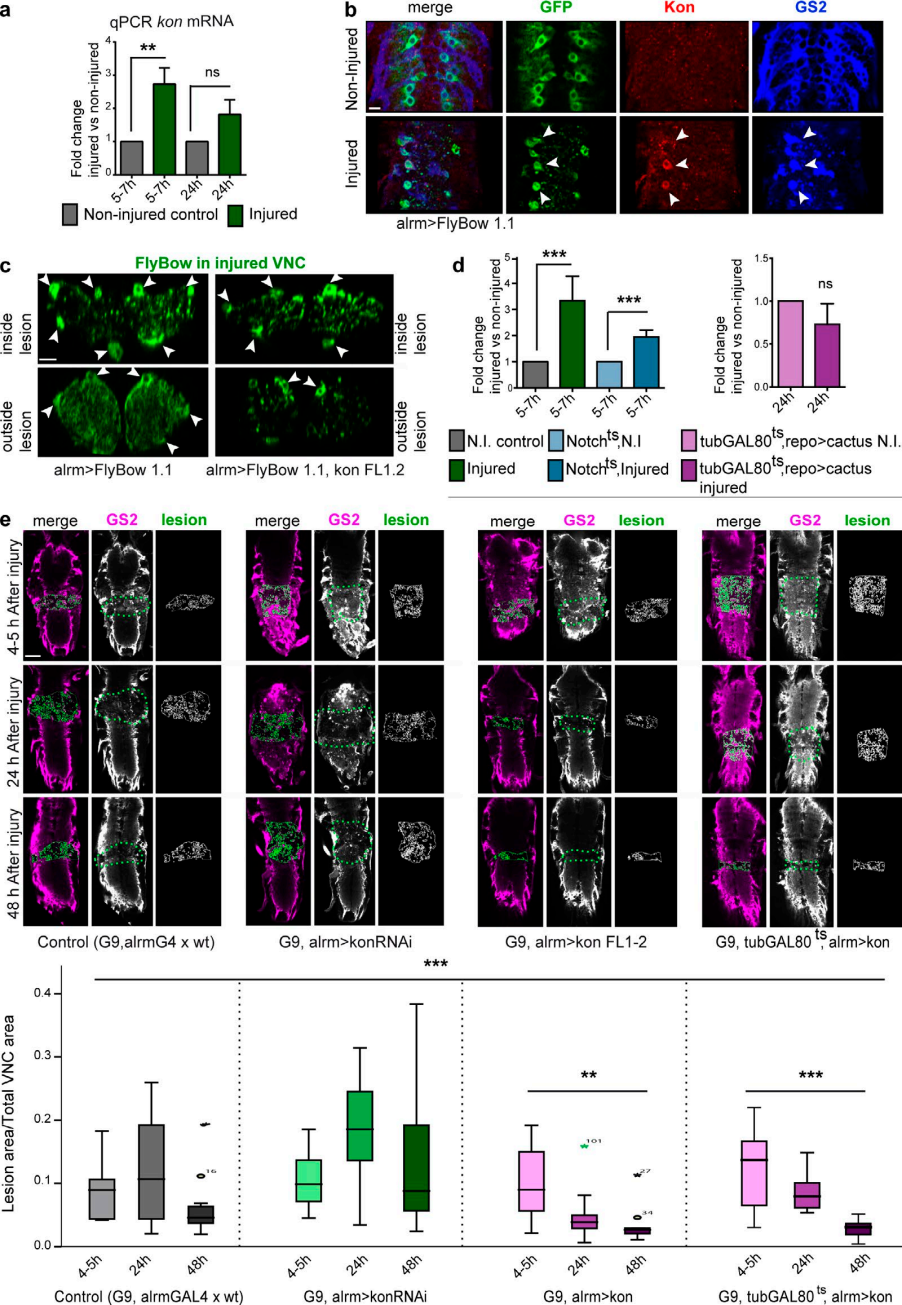
**Kon is required in glia for CNS repair.** All injured samples except in panels b and c have the G9 GFP reporter in their genotype. (a) Genotype: *G9/^+^.* qRT-PCR of larval dissected CNSs: crush injury caused the up-regulation of *kon* transcript levels at 5–7 h after injury, followed by a homeostatic decrease by 24 h. One-way ANOVA: *, P < 0.01; post hoc Sidak paired tests injured vs. noninjured: **, P < 0.01. ns, not significant. (b) Crush injury up-regulated Kon in *alrmGAL4>mCD8GFP* GS2^+^ NG (arrowheads). (c) Crush injury induced a change in cell shape, as the glial *alrmGAL4>FlyBow1.1* fine projections became thicker within the lesion. Overexpression of *kon* with *alrmGAL4* had the same effect. Arrowheads indicate NG nuclei and soma as landmarks. (d) qRT-PCR showing *kon* mRNA levels: upon injury, *kon* expression does not increase as much in *Notch^ts^*; *G9* mutants as in controls. N.I., not injured. One-way ANOVA: ***, P < 0.0001; post hoc Holm–Sidak, injured control vs. noninjured control: ***, P < 0.0001; *Notch^ts^*; *G9* injured vs. *Notch^ts^*; *G9* noninjured control: ***, P < 0.001. *kon* expression fails to increase upon injury in larvae overexpressing *cactus* in glia (*G9*; *tubulinGAL80ts*, *repoGAL4>UAScactus*), Student’s *t* test: P = 0.089. (e) Crush injury in control larvae caused lesion expansion followed by shrinkage. *kon* knockdown in *alrmGAL4* NG prevented wound shrinkage; *kon* overexpression in *alrmGAL4* NG prevented lesion expansion and enhanced lesion repair compared with controls; temporal restriction of *kon* overexpression to after injury (shifted to 30°C immediately after injury) still prevented wound expansion and promoted repair. Asterisks over dataset denote Kruskal–Wallis, ***, P < 0.001; in UASkonFL, multiple comparison Bonferroni correction, **, P < 0.01; *, P < 0.05. (a and d) Sample types are dissected CNS, *n* = 20 CNS per genotype per replicate, three replicates per genotype. (e) Sample types are larval VNCs, *n* = 8–20. For further details, see Table S1; >, GAL4/UAS. Bars: (b and c) 10 µm; (e) 50 µm.

We next asked whether Kon might be involved in the GRR to crushing injury. As injury induces glial proliferation, and Kon can induce glial proliferation, we tested whether *kon* expression was altered upon injury. Using qRT-PCR, we observed a 2.7-fold increase in *kon* mRNA levels at 5–7 h after injury (Fig. 7 a). At 24 h after injury, *kon* mRNA levels were still higher than in intact controls but lower than earlier on (Fig. 7 a). Thus, injury caused the up-regulation of *kon* expression, and mRNA levels decreased over time, implying an underlying homeostatic mechanism. Most dramatically, whereas Kon protein was barely detectable in intact larvae, injury induced the up-regulation of Kon in *alrm>FlyBow1.1* GS2^+^ NG (Fig. 7 b). This demonstrates that injury induces the up-regulation of Kon in NG cells. Crush injury and *kon* overexpression also induced a similar change in cell morphology. In controls, outside the lesion, NG had cell bodies surrounding the neuropil and very fine projections both enwrapping the neuropil and extending into the neuropil (Fig. 7 c). Upon crush injury, the cell bodies were clearly visible, but the projections were thicker and sparsely distributed (Fig. 7 c). Overexpression of *kon* in NG (*alrmGAL4>UASFlyBow*, *UASkon*) had the same effect, both within and outside the lesion (Fig. 7 c). These data show that injury induces the up-regulation of Kon levels in glia and suggest that Kon mediates injury-induced glial activation.

Our genetic epistasis analysis had shown that *kon* functions downstream of *Notch.* Thus, we asked whether the injury-induced up-regulation of *kon* expression depended on Notch signaling. *Notch^ts1^* mutants are heat sensitive, i.e., Notch function is normal at 18°C but lost at 30°C. Larvae were bred at 18°C, moved to 30°C 24 h before injury, and kept at 30°C for a further 5–7 h after injury. *kon* transcript levels were still up-regulated under this regimen in *Notch^ts^* mutants, but considerably less than in intact controls (Fig. 7 d). Thus, the injury-induced up-regulation of *kon* during the GRR depends on Notch.

CNS injury induces the TNF-dependent nuclear translocation of the nuclear factor (NF)-κB homologue Dorsal, which promotes glial proliferation (Kato et al., 2011). Thus we asked whether during the GRR, *kon* might be influenced by Dorsal. We overexpressed in glia the Dorsal inhibitor *cactus*, which prevents its nuclear translocation and gene activation. *Cactus* overexpression was restricted to the GRR only, for 5–7 h after injury (using *tubulinGAL80^ts^*; *repoGAL4>UAScactus*), and this prevented the injury-induced up-regulation of *kon* expression (Fig. 7 d). This shows that *kon* up-regulation in the GRR also depends on Dorsal.

To test whether the injury-induced increase in Kon expression was functionally relevant, we asked whether altering Kon levels affected lesion repair. *kon* knockdown in NG (*G9*, *alrmGAL4>UASkonRNAi^106680^*) resulted in an increase in lesion size at 24 h, and most remarkably, prevented the subsequent lesion shrinkage, as by 48 h the wounds remained larger than at 4 h (Fig. 7 e). Remarkably, an equivalent phenotype is caused by *NG2* knockout in mice, as the lesion does not shrink either (Kucharova et al., 2011). Conversely, *kon* overexpression in NG (*G9*, *alrmGAL4>UASkon*) prevented lesion expansion at 24 h, and most dramatically, resulted in virtually complete repair by 48 h (Fig. 7 e). To test whether this remarkable repair was caused by the overall increase in NG number with *kon* overexpression, or to enhanced GRR, we overexpressed *kon* only during the GRR, by shifting to 30°C at injury (*G9*, *tubulinGAL80^ts^*, *alrmGAL4>UASkon*)*.* Temporal restriction of *kon* overexpression in Pros^+^ alrm^+^ NG was sufficient to induce repair, as the lesion had shrunk by 24 h after injury and was fully repaired by 48 h (Fig. 7e). These data demonstrate that Kon is required in NG for, and can promote, CNS repair.

## Discussion

We have shown that the *Drosophila NG2* homologue *kon* is expressed and required in NG for glial proliferation, activation, and onset of glial differentiation and is necessary and sufficient for CNS injury repair. Kon functions within a gene network involving Notch and Pros in glia, and analogous genetic relationships may exist in the OL cell lineage in the mammalian CNS.

**Figure 8.**
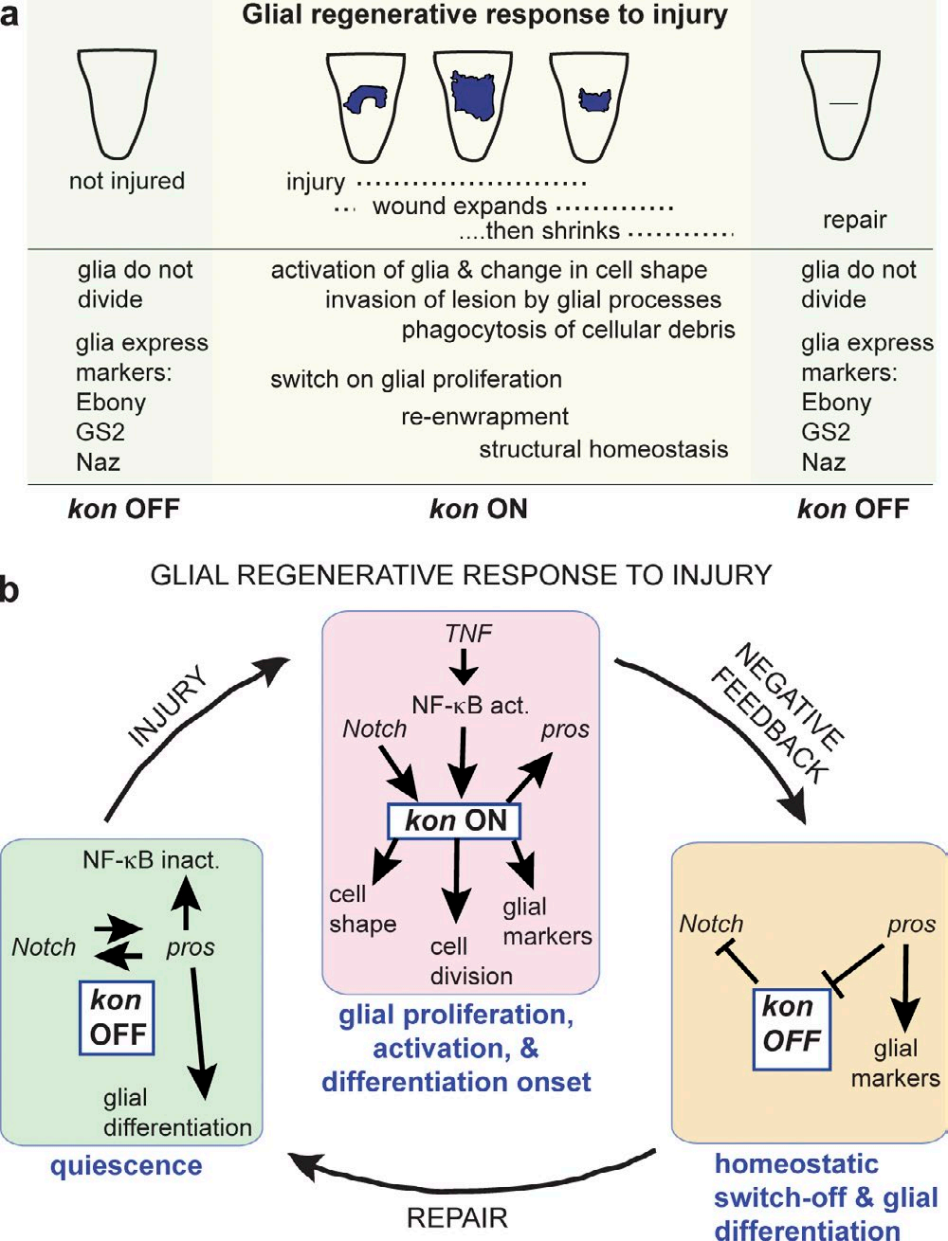
**Kon promotes CNS repair together with Notch, Pros, and NFκB.** (a) Natural progression of injury. (b) Quiescence: Kon is normally virtually off, and NG are quiescent but kept ready to divide. Injury: Kon is switched on, and Kon downstream of Notch provokes glial proliferation, also enhanced by NFκB. Kon also induces glial cell shape changes and the expression of glial markers (activation and onset of glial differentiation). Negative feedback: After cell division, Kon represses Notch signaling, and Pros represses *kon* expression, altogether switching Kon off. These negative feedback loops limit the lifetime of Kon and restore cell number and cell shape homeostasis, essential for repair.

We found *kon* on in proliferating glia, in development and in response to injury, and off in quiescent and differentiated larval glia (Fig. 8 a). Our genetic epistasis analysis revealed that *kon* is regulated by two feedback loops (see also Fig. 5 d). (1) *Notch* activates *kon* expression, and *kon* represses *Notch.* The Notch–Kon feedback loop promotes glial proliferation, constrains the lifetime of *kon* expression, and restores cell number homeostasis. (2) *kon* activates *pros*, and *pros* represses *kon* expression. Kon regulates the expression of the glial differentiation markers Repo, Pros, Ebony, GS2, and Naz, and interfering with Kon function altered glial shape, but Kon was barely detectable in quiescent and differentiated glia, and overexpression of *kon* did not up-regulate glial markers in Pros-negative glia. Thus, Kon is required for glial activation and the onset of glial differentiation but not for maintenance of the differentiated state, which depends on Pros (Griffiths and Hidalgo, 2004; Kato et al., 2011). Kon activates Pros, which inhibits *kon* expression, preventing further glial cell division, and as Kon is down-regulated, glial differentiation is maintained by Pros. Thus, the Kon–Pros feedback loop enables the transition from glial proliferation to differentiation and restores cell shape homeostasis.

Kon drives the GRR to injury as follows (Fig. 8 b). In the intact larva, Pros^+^ NG are quiescent in G1 (Griffiths and Hidalgo, 2004; Kato et al., 2011) and have both Notch and Pros, and Kon in low levels (i.e., Kon off). Pros-negative NG are in G0 and cannot enter the cell cycle (Griffiths and Hidalgo, 2004). At this stage, the Pros^+^ NG appear differentiated and interact with synapses, enwrap axons, and express Ebony, GS2, and Naz (Kato et al., 2011; Stork et al., 2014; Peco et al., 2016). Injury triggers a sharp increase in Kon levels in Pros^+^ NG (i.e., *kon* on), NG cell division, and activation. The up-regulation of *kon* upon injury depends on Notch and NF-κB/Dorsal. Similarly, in demyelinating lesions in mammals, Notch and NG2 are also up-regulated in NG2^+^ OPCs (Genoud et al., 2002; Stidworthy et al., 2004). NFκB/Dorsal are activated upon injury in glia, in both mammals and flies, by TNF signaling, and together, Notch and NFκB/Dorsal activate glial proliferation upon injury (Kato et al., 2011). We have shown that they achieve so by up-regulating Kon. Overexpression of Kon also induced dramatic changes in cell shape, suggesting that Kon induces glial activation. During activation, NG enlarge their cytoplasms and become phagocytic (Kato et al., 2011). Kon positively regulates multiple glial markers—Ebony, GS2, and Naz—at the onset of glial differentiation. After cell division, *kon* represses *Notch*, thus restricting glial proliferation. Because Kon depends on Notch, this negative feedback loop limits Kon’s lifetime after injury. Kon also activates *pros*, and as Pros is up-regulated, it maintains the expression of glial cell markers such as Ebony and GS2 and promotes axonal enwrapment (Kato et al., 2011). Pros also represses *kon* expression, preventing cell-cycle reentry. This negative feedback loop enables glial differentiation after injury. We demonstrated that down-regulation of Kon in Pros^+^ NG prevented repair, and up-regulation of Kon in Pros^+^ NG enhanced repair. To conclude, Kon controls structural homeostasis: the Kon–Notch feedback loop regulates glial cell number, and the Kon–Pros feedback loop regulates the transition from proliferating to quiescent and differentiated glia. It is a homeostatic mechanism that enables repair while preventing an uncontrolled response.

*Drosophila* neuropil glia share features of mammalian astrocytes, NG2^+^ OPCs, and OLs. The alrmGAL4^+^ NG cells are also known as astrocytes, as they invade the neuropil, establish contact with synapses, and express Ebony, EAAT1, and GS2, which are involved in neurotransmitter reuptake (Doherty et al., 2009; Stork et al., 2014; Peco et al., 2016). In the larval VNC, alrmGAL4^+^ cells are the Pros^+^ NG (Kato et al., 2011), which require *kon* for the GRR. In mammals, the *pros* homologue *Prox1* is not expressed in astrocytes or neurons; instead, it is expressed in both OPCs and OLs and is enriched in OLs, where it is required for OL differentiation (Cahoy et al., 2008; Kato et al., 2015). The *kon* homologue NG2 is expressed in pericytes, microglia, and OPCs, but it is not expressed in OLs or astrocytes (Cahoy et al., 2008). Accordingly, the alrmGAL4^+^ Pros^+^ glia would correspond to *Drosophila* NG2 cells. In mammals, NG2 cells also interact closely with synapses and express neurotransmitter receptors, and before the discovery of NG2, astrocytes and OPCs were not unambiguously identified (Raff et al., 1983; Trotter et al., 2010; Zuo and Nishiyama, 2013). More recently, molecular profiling has established that astrocytes and OPCs are distinct cell types: astrocytes do not express Prox1 or NG2; OPCs express Prox1 and NG2, and differentiated OLs express increased Prox1 but not NG2 (Cahoy et al., 2008). *Drosophila* alrmGAL4^+^ NG express Pros and, upon injury, Kon. In *Drosophila*, all NG contribute to the ensheathment of the neuropil and of axons within the neuropil (Kato et al., 2011). In mammals, NG2^+^ OPCs do not ensheath axons (Franklin and ffrench-Constant, 2008). OLs do, and they express Prox1 and GS, and Prox1 is required for OL differentiation (Domercq et al., 1999; Rubin and Kessaris, 2013; Kato et al., 2015). In *Drosophila*, GS2 is in glial processes enwrapping the neuropil and nerves, and Pros is required for axonal enwrapment (Kato et al., 2011). Together, these facts reveal shared features between *Drosophila* Pros^+^ NG glia and the mammalian NG2 cells/OPCs and OLs.

CNS repair in *Drosophila* critically depends on Kon in neuropil glia. In mammals, NG2^+^ cells are the main glial cell type to divide upon injury and have a prominent proregenerative response to injury (Franklin and ffrench-Constant, 2008; Trotter et al., 2010; Zuo and Nishiyama, 2013). In both mice and flies, injury induces the up-regulation of NG2 and Kon levels in glia (Kucharova et al., 2011; and this work). As in *Drosophila*, NG2, Notch1, and the Pros homologue Prox1 coexist in mammalian OPCs (Wang et al., 1998; Genoud et al., 2002; Rubin and Kessaris, 2013; Kato et al., 2015), and as Prox1 levels rise, OPCs differentiate into OLs, which lack NG2 and have high Prox1 levels (Kato et al., 2015). Thus, as in fruit flies, the transition from proliferating OPCs to differentiated OLs is characterized by a decrease in NG2 levels and an increase in Prox1. Prox1 could be the key factor functionally related to NG2 promoting OL differentiation to drive remyelination after injury.

Our work has revealed a key functional link between Notch, Kon, and Pros for CNS repair, which could be evolutionarily conserved. *Drosophila* genetics must be exploited further for the understanding of CNS repair, with important implications for manipulating stem cells and glial progenitors in humans.

## Materials and methods

### Genetics

Experiments were performed in mid-third-instar larvae at 98–104 h after egg laying (AEL) or at the wandering stage (120 h AEL) at 25°C, unless otherwise indicated. Genotype: wild-type is *yw* or *Oregon R*. Reporter lines: (a) *p12xSu*(*H*)*bs-lacZ* (gift of M. Okabe, The Jikei University School of Medicine, Tokyo, Japan), reporter for Notch signaling (Kato et al., 2011); (b) *G9*, protein-trap line driving expression of GFP in all axons (gift of W. Chia, Temasek Life Sciences Laboratory, Singapore, Singapore; Kato et al., 2011); (c) *w; UAShistoneYFP*, drives YFP in nuclei; (d) *w; UAS-Flybow1.1* (gift from I. Salecker, The Francis Crick Institute, London, England, UK). Mutants: (a) *pros^S044116^*/*TM6B* (FBal0082320) and *pros^voila1^*/*TM6B* (FBti0010694), hypomorphic larval viable alleles (Kato et al., 2011); (b) *Notch^ts1^*/*FM7(sn^+^)actGFP* (FBal0012887), temperature-sensitive loss-of-function allele (gift of A. Martinez-Arias, University of Cambridge, Cambridge, England, UK); (c) *w; kon^C452^/CyO Twi-GFP* (FBal0217365; gift of F. Schnorrer, Max Planck Institute of Biochemistry, Martinsried, Germany). Overexpression and knock-down GAL4 drivers: (a) *w;; repoGAL4*/*TM6B*, drives expression in all glia except midline glia; (b) *w;; elavGAL4*, drives expression in all neurons; (c) *w; alrmGAL4* (on second or third chromosomes; gift of M. Freeman, University of Massachusetts Medical School, Worcester, MA), drives expression in Pros^+^ NG in the larval VNC; (d) *NP6520*, drives GAL4 expression in Pros-negative NG. GAL80: (a) *tubulinGAL80^ts^*, represses GAL4 at 18°C but not at 25°C; (b) CyO-GAL80 was used to maintain stocks carrying both GAL4 and UAS transgenes. UAS lines: (a) *w; UASNotch^ICD^myc*/*TM6B* (gift of A. Martinez-Arias); (b and c) *w; UAS-HA-kon-FL1-2* and *w;; UAS-HA-kon-FL4-1* (gift of F. Schnorrer); (d) *UASkonRNAi^v106680^* (VDRC); (e) *w; UASpros-k* (gift of F. Matsuzaki, Center for Developmental Biology, RIKEN, Kobe, Japan); (f) *UAS-pros RNAi: y v; P{TRIP.JF02308}attP2/TM6B* (Bloomington Drosophila Stock Center); (g) *w; UAScactus-3xHA* (FlyORF; Bischof et al., 2013). Genotypes of interest were identified by the lack of *TM6B Tb^—^*, *CyO-DfdYFP*, or *FM7(sn^+^)actGFP* balancers. Stocks bearing combinations of mutants, reporters, GAL4, or UAS were generated by conventional genetics. For full genotypes and samples sizes, see Table S1.

### Temporal control of gene expression in larval stages and BrdU incorporation

Temperature-sensitive *Notch^ts1^* embryos were kept at the permissive temperature (18°C for 48 h) and shifted to the restrictive temperature (30°C) from L1 to wandering-third-instar larval stage, when experiments were performed. Only male larvae were used.

To drive gene expression in larval stages only, we used the temperature-sensitive GAL4 repressor GAL80^ts^, driven by the general *tubulin* promoter, in *tubGAL80^ts^* flies (Bloomington Drosophila Stock Center), as previously described (Kato et al., 2011). Flies of three genotypes, (a) *tubGAL80^ts^*; *repoGAL4*, (b) *tubGAL80^ts^*; *elavGAL4*, and (c) *tubGAL80^ts^*; *alrmGAL4*, were generated by conventional genetics and crossed to *w; UAS-HA-kon-FL1-2* flies. First-instar larvae were raised at 18°C for 44–48 h AEL, then shifted to 30°C and fixed 70–72 h later. For overexpression of *kon* combined with BrdU incorporation, mid-third-instar larvae (157–159 h AEL at 18°C) were placed at 30°C; after 1 h, BrdU food (1 mg/ml) was added, and after 6 h, larvae were changed to fresh food without BrdU, kept at 30°C for another 18 h, and then dissected. For BrdU incorporation in *G9* injured larvae, larvae were injured at 74–76 h AEL at 25°C, fed with BrdU food (1 mg/ml) for 6 h, transferred to soft food plates, and dissected 18 h later.

### BrdU incorporation in larvae

For overexpression of *kon* in larvae combined with BrdU incorporation, flies bearing *tubGAL80^ts^* and GAL4 drivers were crossed to *w; UAS-HA-kon-FL1-2* flies and raised at 18°C until mid-third-instar larval stage (157–159 h AEL at 18°C), when larvae were shifted to 30°C. After 1 h, BrdU food (1 mg/ml) was added; after 6 h, flies were changed to fresh food without BrdU and kept at 30°C for another 18 h, and then dissected. For BrdU incorporation in *G9* injured larvae, larvae were injured at 74–76 h AEL at 25°C, fed with BrdU food (1 mg/ml) for 6 h, transferred to soft food plates, and dissected 18 h later.

### Crushing injury to the larval ventral nerve cord

Early-third-instar larvae (74–76 h AEL at 25°C), bearing the *G9* CNS or *alrmGAL4>UASFlyBow1.1* reporters, were anesthetized on ice for 7 min. Next, each larva was placed ventral side up in a cold Sylgard plate under a fluorescent dissecting microscope, with the anterior–posterior axis horizontal. With one hand, the larva was held in place, pressing gently using forceps. With the other hand, finer forceps were used to pinch the VNC very carefully, aiming for the abdominal VNC, without producing a complete break. The injured larva was moved to a “recovery plate” containing soft food (80 ml final volume: 5 g sugar, 0.2 g salt, 5 g yeast, 0.3 g agar, 2.5 g flour, and 10 ml fruit juice and tap water), until dissection was required. The features of the glial regenerative response to injury—progression of lesion size, induction of glial proliferation, and glial activation—were analyzed in crush-injured specimens and compared with the features previously described for stabbing injury (Kato et al., 2011; Kato and Hidalgo, 2013).

Temporal restriction of *Notch* loss of function during the GRR was performed using the Notch^ts1^ allele. *Notch^ts1^*/*FM7(sn^+^)actGFP* females were crossed to *G9* males, and progeny were allowed to develop at 18°C for 120 h. They were then placed at the restrictive temperature, 30°C, for 24 h. Larvae were sexed, and *Notch^ts^* males were injured and returned to 30°C for 5 h before being dissected and treated for qRT-PCR.

Temporal restriction of Dorsal inhibition during the GRR was performed by overexpressing *cactus* in *tubulinGAL80^ts^*; *repoGAL4* flies crossed to *UAScactus* flies. Progeny were allowed to develop at 18°C for 168 h, when they were injured at the third-instar larval stage. Immediately after injury, they were placed at 30°C and were dissected and treated for qRT-PCR 24 h later.

Temporal restriction of *kon* overexpression during GRR was performed by crossing *G9, tubulinGAL80^ts^*, *alrmGAL4* flies to *UASkonFL1-2* flies. Progeny were raised at 18°C for 144 h; at this point (as third-instar larvae) they were injured and maintained at 30°C until dissection at 4–5, 24, or 48 h, then were fixed and stained. Images were acquired with confocal microscopy. The wound area was measured as indicated in “Wound area measurement.”

### qRT-PCR

qRT-PCR was performed according to standard methods. For developmental profiles, 2-h egg collections were performed at 25°C, whole embryos were harvested 20 h after egg laying (AEL) and dechorionated, and the CNS was dissected from second-instar larvae (L2) at 65 h AEL, third-instar larvae (L3) at 96 h AEL, and pupae 0–12 h after puparium formation. For all other experiments, wandering-third-instar larvae (120 h AEL at 25°C) were used, except that *Notch^ts^* embryos were grown at the permissive temperature of 18°C for 48 h, shifted to 30°C at first-instar larval (L1) stage, and mutant males were harvested at the wandering-third-instar larval stage; *tubulinGal80^ts^;repoGAL4>UAS pros RNAi* samples were grown at 18°C for 144 h before being shifted to 30°C. Samples were placed immediately into TRI reagent (#AM9738; Ambion) and frozen at −80°C. Total RNA was extracted from 20 embryos or 20 dissected larval or pupal CNSs, using TRI and following manufacturers’ instructions. cDNA was synthesized from 300 ng total RNA using the GOScript reverse transcription system (#A5001; Promega) using random primers, diluted threefold for qPCR reactions, and 2 µl used per reaction. Controls without reverse transcription were run alongside cDNA reactions. Transcript levels were determined in triplicate for each sample using SensiFAST Hi-ROX SYBR GREEN (#BIO-92020; Bioline USA) run on an ABI Prism 7000 sequence detection system. The reference gene for all experiments was *RpL32* (Ling and Salvaterra, 2011), except for injury experiments, in which *gapdh2* was used, as it had previously been shown to be unaltered in injury (Schuster and Smith-Bolton, 2015). Primers used were as follows: (a) RpL32qPCRF: 5′-AAGCGGCGACGCACTCTGTT-3′; (b) RpL32qPCRR: 5′-GCCCAGCATACAGGCCCAAG-3′; (c) GAPDH2F qPCR: 5′-GTGAAGCTGATCTCTTGGTACGAC-3′; (d) GAPDH2R qPCR: 5′-CCGCGCCCTAATCTTTAACTTTTAC-3′; (e) *Kon*qPCR (Ex10-11) F3: 5′-CCCAAGCGATTTCTTTACCA-3′; and (f) *Kon*qPCR (Ex10-11) R3: 5′-TTGATGGAAACGGGAATTGT-3′.

To obtain fold change values using the 2-ΔΔCt method (Livak and Schmittgen, 2001), the count threshold (Ct) value of *Rpl32* or *Gapdh2* was subtracted from the Ct value of *Kon* (ΔCt). All values were then normalized to a calibrator. For the developmental profile, the calibrator was *kon* mRNA embryonic levels; for the other experiments, the calibrators were the control genotype (ΔΔCt). At least three independent biological replicates were performed per experiment. Statistical analyses were performed on the ΔCt values to the control gene (*Rpl32* or *Gapdh2*) using Graphpad6 and either Student’s *t* test or one-way analysis of variance (ANOVA) followed by either post hoc Dunnett’s or Sidak’s multiple comparison corrections. For all statistics details, see Table S1.

### Immunohistochemistry, BrdU detection, and in situ hybridization

In situ hybridizations to *kon* mRNA were performed with digoxigenin-labeled antisense RNA probes and alkaline phosphatase detection (Ambion kit), using cDNA plasmid LD31354 (Drosophila Genomics Resource Center), linearized with EcoRI and transcribed with SP6 RNA polymerase. For BrdU incorporation, the dissected CNSs were treated with 2 M HCl for 20 min at RT before incubation with anti-BrdU. Incubation with primary antibodies was performed at 4°C overnight, and fluorescent-conjugated secondary incubation was performed at RT for 1.5 h. Primary antibodies were at the following dilutions: (a) mouse anti-Repo, 1:250 (Developmental Studies Hybridoma Bank); (b) guinea pig anti-Repo, 1:1,000 (gift from B. Altenhein, University of Mainz, Mainz, Germany); (c) rabbit anti-Ebony, 1:250 (gift of S. Carroll, University of Wisconsin, Madison, WI); (d) mouse anti–glutamine synthetase, 1:250 (EMD Millipore); (e) mouse anti-Pros, 1:250 (Developmental Studies Hybridoma Bank); (f) mouse anti–β-galactosidase, 1:200 (Developmental Studies Hybridoma Bank); (g) mouse anti-BrdU, 1:250 (Developmental Studies Hybridoma Bank); (h) mouse anti–FasII ID4, 1:250 (Developmental Studies Hybridoma Bank); (i) rabbit anti-*kon*, 1:1,000 (gift of F. Schnorrer); and (j) rabbit anti-Nazgul, 1:250 (gift of B. Altenhein). Alexa Fluor 488–, 546–, 660–, or 647–conjugated secondary antibodies (Molecular Probes) were used.

### Microscopy, imaging, and quantitative analysis

#### Microscope image acquisition

Fluorescent images were acquired using a ZEISS LSM 710 Confocor3 laser scanning confocal microscope, with 25× or 40× oil immersion objectives, numerical aperture 0.8 and 1.3, respectively, at 512 × 512- or 1,024 × 1,024-pixel resolution, with 0.51- or 0.96-µm steps, throughout the entire larval VNC. Specimens were fixed and mounted in Vectashield Antifade Mounting Medium (H-1000; Vector Laboratories) at RT. The fluorochromes used were Alexa 488, 546, and 633, directly conjugated to secondary antibodies, and membrane-tethered GFP and YFP, fused to histone. Stacks of images spanning the entire VNC were acquired using ZEN software (ZEISS). Bright-field images were acquired using an Axioplan 2 microscope (ZEISS) using Nomarski optics and an AxioCam camera, with 63× oil immersion objective, numerical aperture of objective 1.4 and oil condenser, adapter 0.63×, and ZEN acquisition software. Stacks of images were processed using ImageJ, and Photoshop and Illustrator (Adobe) were used to process images and compile figure plates.

#### Cell counting

(a) Repo^+^ cells and HistoneYFP^+^ cells were counted automatically throughout the entire abdominal CNS in 3D, throughout stacks of confocal images, using the DeadEasy Larval Glia ImageJ plugin (Kato et al., 2011; Forero et al., 2012), except in *repoGAL4>UASKonRNAi,* where cells were counted manually with the aid of the ImageJ Cell Counter macro. DeadEasy identifies cells based on volume and pixel intensity, in 3D. Mouse anti-Repo antibody (Developmental Studies Hybridoma Bank) was used. To delimit the abdominal VNC, T3 exit glia cells were used as a landmark. (b) Abdominal Pros^+^ NG were counted manually in *alrmGal4>UAS-FlyBow1.1* flies using mouse anti-Pros, with the aid of the ImageJ Cell Counter macro. To delimit the abdominal VNC, the proliferating area defined by Pros^+^ neuroblasts was used as a landmark. (c) BrdU^+^ NG were identified with anti-Ebony and mouse anti-BrdU (Developmental Studies Hybridoma Bank) and counted manually with the aid of the ImageJ Cell Counter macro. To delimit the abdominal VNC, neuroblast proliferation detected by BrdU was used as a landmark.

#### Wound area measurement

Crush injury lesions were identified in G9, anti-GS2 stained samples as devoid of fluorescent signal, with the aid of ImageJ macros. For each sample, the lesion area was first selected from the whole stack of confocal images as the optical section with the largest lesion area. Using ImageJ (Fiji), brightness was adjusted to better see the lesion, and the wound was outlined with the region-of-interest macro. Adjusted mean threshold was applied to automatically set a signal threshold, and this was run throughout the entire stack of images to determine the lesion size in each optical section. The macros together identified the area devoid of signal and provided the area measurement. The largest lesion area per specimen was selected and normalized over total VNC area relative to the posterior end of the optic lobes.

### Statistical analysis

Statistical analyses were performed using SPSS Statistics and GraphPad Prism software, and significance set at 95% confidence. For data with normal distributions and equal variances according to Levene’s test, one-way ANOVA was applied to compare means, followed by post hoc Dunnett’s or Sidak’s multiple comparisons to a fixed control, or otherwise a Bonferroni multiple comparisons test. Welch’s ANOVA was used when samples were distributed normally but did not pass Levene’s test, followed by post-hoc Games–Howell. Student’s *t* test was used if comparing only two normally distributed sample types. For data in which distributions were not normal, the nonparametric Kruskal–Wallis test was applied for multiple comparisons, followed by a post hoc Dunn multiple comparisons correction to a fixed control, and Mann–Whitney *U* test when only two sample types were compared. Reproducibility for experiments performed in larvae, in vivo, was determined by treating control and experimental samples at the same time, under the same conditions, and by increasing sample sizes through repeats. For qRT-PCR, all experiments were repeated at least three times, with three different biological replicates. For genotypes, sample sizes, tests, and p-values, see Table S1.

### Online supplemental material

Fig. S1 shows that *kon* overexpression changes glial shape. Fig. S2 shows regulation of Pros and Ebony by Kon-tiki. Table S1 shows statistical analysis details. Online supplemental material is available at http://www.jcb.org/cgi/content/full/jcb.201603054/DC1.

## Supplementary Material

Supplemental Materials (PDF)

Table S1 (Excel file)

Table S1
