# Supplementary material for: Molecular mechanism of central nervous system repair by the Drosophila NG2 homologue kon-tiki
Source: J Cell Biol. 2016 Aug 29;214(5):587–601. doi: 10.1083/jcb.201603054 (PMC5004445; doi:10.1083/jcb.201603054)
Supplement: Supplemental Materials (PDF) [file JCB_201603054_sm.pdf]

Losada-Perez et al., http://www.jcb.org/cgi/content/full/jcb.201603054/DC1

THE JOURNAL OF CELL BIOLOGY

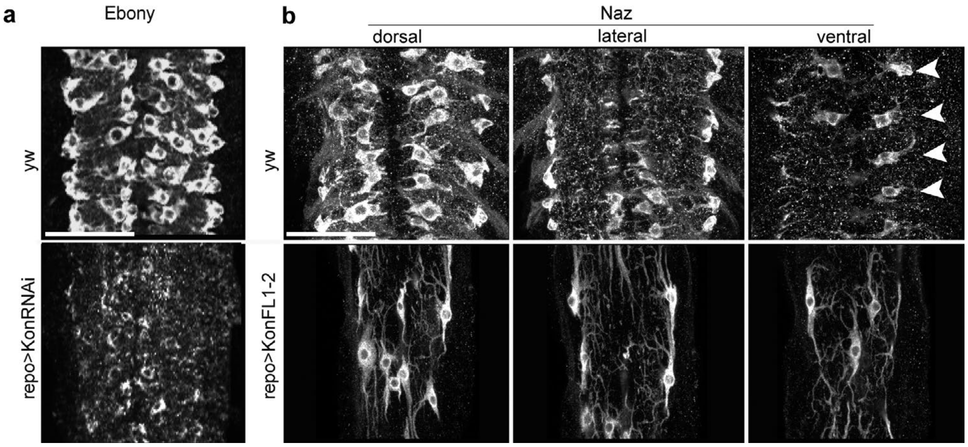

Figure S1. ***kon* overexpression changes glial shape.** (a) *kon* knockdown with *repoGAL4* dramatically down-regulates Ebony. (b) *kon* overexpression with the stronger line, *UASkonFL1-2*, results in dramatic changes in cell shape, seen here with anti-Naz. Notice that the glia have extended, star-shaped filopodia; however, this could simply be a secondary deformation of the extremely long VNCs in this genotype. Bars, 50 µm.

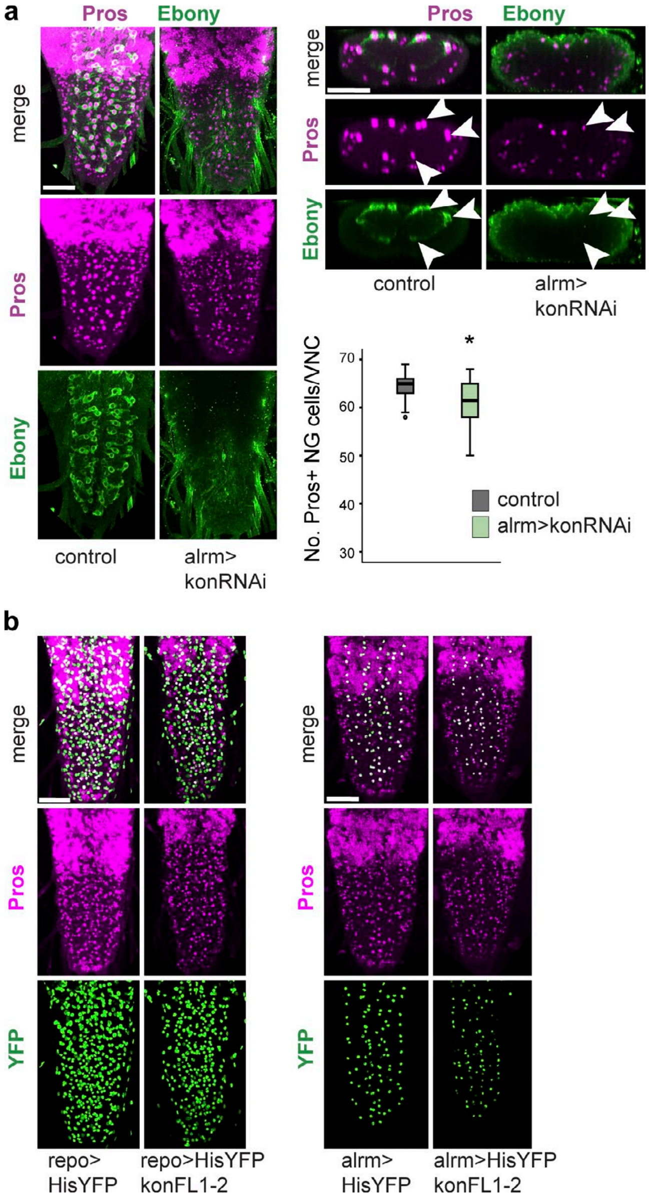

Figure S2. **Regulation of Pros and Ebony by Kon-tiki.** (a) *kon* RNAi down-regulates Pros and Ebony (arrowheads) and decreases Pros+ NG cell number. Horizontal views on the left, transverse on the right. Mann-Whitney *U* test: *, P < 0.05. (b) Overexpression of Kon either in all glia (with *repoGAL4*) or in Pros+ NG (with *alrmGAL4*) does not affect Pros. Glia are visualized with a HistoneYFP reporter. >, GAL4/UAS. (a) Sample types are larval VNCs, *n* = 10–21. For further details, see Table S1. Bars, 50 µm.

Provided in a separate Excel file is Table S1, showing statistical analysis details.
