# Supplementary material for: Molecular mechanism of central nervous system repair by the Drosophila NG2 homologue kon-tiki
Source: J Cell Biol. 2016 Aug 29;214(5):587–601. doi: 10.1083/jcb.201603054 (PMC5004445; doi:10.1083/jcb.201603054)
Supplement: Table S1 [file jcb.201603054_index.html]

Supplement to Molecular mechanism of central nervous system repair by the Drosophila NG2 homologue kon-tiki | JCB

## 

**Files in this Data Supplement:**

- Supplemental Materials (PDF)
- Table S1 (Excel file)
